# *Atg5* Supports *Rickettsia australis* Infection in Macrophages *In Vitro* and *In Vivo*

**DOI:** 10.1128/IAI.00651-18

**Published:** 2018-12-19

**Authors:** Jeremy Bechelli, Leoncio Vergara, Claire Smalley, Tetyana P. Buzhdygan, Sean Bender, William Zhang, Yan Liu, Vsevolod L. Popov, Jin Wang, Nisha Garg, Seungmin Hwang, David H. Walker, Rong Fang

**Affiliations:** a Department of Pathology, University of Texas Medical Branch at Galveston, Galveston, Texas, USA; b Center for Biomedical Engineering, University of Texas Medical Branch at Galveston, Galveston, Texas, USA; c Department of Microbiology and Immunology, University of Texas Medical Branch at Galveston, Galveston, Texas, USA; d High School Student Summer Research Program, University of Texas Medical Branch at Galveston, Galveston, Texas, USA; e Immunobiology and Transplant Science Center, Houston Methodist Research Institute, Houston, Texas, USA; f Department of Pathology, University of Chicago, Chicago, Illinois, USA; g Department of Biological Sciences, Sam Houston State University, Huntsville, Texas, USA; Washington State University

**Keywords:** *Atg5*, IL-1β, *Rickettsia australis*, autophagosomes, mouse macrophages

## Abstract

Rickettsiae can cause life-threatening infections in humans. Macrophages are one of the initial targets for rickettsiae after inoculation by ticks. However, it remains poorly understood how rickettsiae remain free in macrophages prior to establishing their infection in microvascular endothelial cells. Here, we demonstrated that the concentration of Rickettsia australis was significantly greater in infected tissues of *Atg5^flox/flox^* mice than in the counterparts of *Atg5^flox/flox^* Lyz-*Cre* mice, in association with a reduced level of interleukin-1β (IL-1β) in serum. The greater concentration of R. australis in *Atg5^flox/flox^* bone marrow-derived macrophages (BMMs) than in *Atg5^flox/flox^* Lyz-*Cre* BMMs *in vitro* was abolished by exogenous treatment with recombinant IL-1β. Rickettsia australis induced significantly increased levels of light chain 3 (LC3) form II (LC3-II) and LC3 puncta in *Atg5*-competent BMMs but not in *Atg5*-deficient BMMs, while no p62 turnover was observed. Further analysis found the colocalization of LC3 with a small portion of R. australis and *Rickettsia*-containing double-membrane-bound vacuoles in the BMMs of B6 mice. Moreover, treatment with rapamycin significantly increased the concentrations of R. australis in B6 BMMs compared to those in the untreated B6 BMM controls. Taken together, our results demonstrate that *Atg5* favors R. australis infection in mouse macrophages in association with a suppressed level of IL-1β production but not active autophagy flux. These data highlight the contribution of *Atg5* in macrophages to the pathogenesis of rickettsial diseases.

## Abstract (precis)

Rickettsiae can cause life-threatening infections in humans. Macrophages are one of the initial targets for rickettsiae after inoculation by ticks.

## INTRODUCTION

Rickettsiae are Gram-negative, obligately intracellular bacteria that can cause potentially life-threatening diseases, which are associated with arthropod transmission. Case-fatality rates were reported to be as high as 65% to 80% in some case series prior to the development of effective antibiotic treatment (1, 2). Misdiagnosis and delayed treatment are often associated with fatal cases (3, 4). The incidence of reported spotted fever group infections in the United States has increased dramatically since the early 21st century (2, 5). The clinical manifestations of rickettsial diseases usually include fever, headache, and rash. Severe cases develop interstitial pneumonia, meningoencephalitis, and multiorgan failure leading to death. Pathogenic rickettsiae are introduced into the skin via an arthropod vector and are then spread via the lymphatics to draining lymph nodes and disseminate hematogenously to infect microvascular endothelial cells systemically, further leading to increased microvascular permeability (6). Although rickettsial infections have been studied for more than 100 years, the underlying mechanisms involved in the pathogenesis of rickettsial diseases remain incompletely understood.

To understand the pathogenic mechanisms of disease caused by spotted fever group rickettsiae after a tick bite, we aimed to investigate the interactions of rickettsiae with their initial target cells. Accumulating evidence from clinical specimens suggests that mononuclear phagocytes, most likely macrophages, are the initial targets for rickettsiae at the onset of the disease: (i) histopathological studies showed that mononuclear cells were infected in the skin inoculation site in a patient infected with Rickettsia parkeri (7), and (ii) immunohistochemical examination demonstrated that the predominant infected cells in the skin lesions of patients with rickettsialpox are CD68-positive mononuclear phagocytes (8). Thus, macrophages most likely serve as the initial targets for rickettsiae in the tick feeding site. Macrophages are key sentinels of the innate immune system and are tasked with detecting and responding to pathogens. Interestingly, the accumulation of both spotted fever and typhus group rickettsiae in macrophage-like cells is closely associated with their virulence (9–12). Typically, virulent R. conorii survives and proliferates in human macrophage-like cells, while nonvirulent R. montanensis is rapidly destroyed (9). Macrophages isolated from mice resistant to R. akari infection show rickettsicidal activity, while those from a susceptible mouse strain are defective in killing rickettsiae (13). R. typhi and R. akari, which are genetically less related to spotted fever group rickettsial species, initiate differential expression levels of proinflammatory cytokines, including interleukin-1β (IL-1β), in macrophages/monocytes (14). However, it is unclear why macrophages cannot efficiently clear the intracellular rickettsiae. These issues and the questions of how rickettsiae survive in macrophages and are subsequently potentially disseminated systemically by macrophages are important to understanding the pathogenesis of rickettsial diseases.

Autophagy is an intracellular, bulk degradation process in which a portion of a cytoplasmic component of the cell is engulfed in double-membrane-bound structures, known as autophagosomes, and subsequently degraded upon fusion with lysosomes (15, 16). Autophagosome formation requires 16 autophagy-related (*Atg*) genes, including *Atg5*, which comprise a ubiquitin-like conjugation system (17). Another ubiquitin-like conjugation system, microtubule-associated protein light chain 3 (LC3-ATG8), is also required in autophagosomal elongation (18). The conversion of a cytosolic truncated form of LC3 (LC3-I) to its autophagosomal membrane-associated, phosphatidylethanolamine-conjugated form (LC3-II), visible as discrete puncta by immunofluorescent analysis, indicates autophagosome formation (19). The protein lipidation system resulting in LC3-II is driven by the *Atg5-Atg12-Atg16* complex acting as an E3 ligase equivalent that facilitates the localized conversion of LC3-I into LC3-II (20), although as the key marker of autophagy, autophagosome-independent LC3 organelles have been described (21). The cargo enveloped by autophagosomes is normally further delivered to autolysosomes for degradation. However, intracellular bacteria have developed a variety of strategies to subvert autophagosomes for the benefit of replication (22, 23). All members of the genus *Rickettsia* are capable of invading host cells and escaping phagosomal vacuoles as quickly as 30 min after infection (24–27). To us, it is important to investigate how *Rickettsia* interacts with the membrane compartments involved in the autophagy pathway in the cytosol of host cells after escaping capture by phagosomes.

Rickettsia australis is genetically related to spotted fever group rickettsiae. The pathological changes seen in R. australis-infected C57BL/6 (B6) mice mimic those seen in human spotted fever rickettsioses, and R. australis-infected B6 mice represent an excellent model of severe rickettsial infection (28, 29). By using this murine model of rickettsioses, we have previously identified the critical roles of cytotoxic T lymphocytes and MyD88 in host protective immunity against rickettsial infection (29, 30). We also mechanistically investigated the *in vivo* contribution of NLRP3, an inflammasome known to mediate the secretion of IL-1β, to host immunity against R. australis using B6-background gene-knockout mice (31). In the present study, we employed B6-background conditional-gene-knockout mice, *Atg5^flox/flox^* Lyz-*Cre* and *Atg5^flox/flox^* mice, in which *Atg5* is deficient mainly in macrophages. We report a new host gene by which rickettsiae manipulate mammalian macrophages to promote the infection *in vivo*. Our results demonstrate that *Atg5* in macrophages favors R. australis infection both *in vitro* and *in vivo*. Our data suggest that *Atg5* supports R. australis infection in macrophages in association with inhibition of the production of IL-1β but not active autophagy flux. Thus, *Atg5* in macrophages appears to contribute greatly to the progression of rickettsial diseases.

## RESULTS

### *Atg5* in macrophages favors R. australis infection *in vivo*.

**FIG 1:**
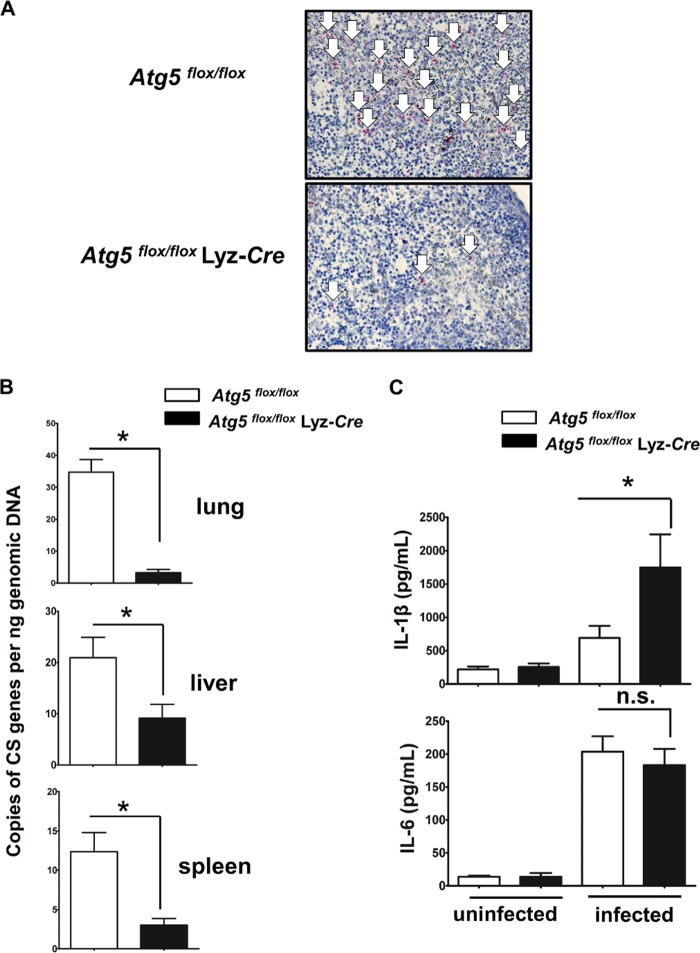
*Atg5* in macrophages favors R. australis infection *in vivo. Atg5^flox/flox^* Lyz-*Cre* and *Atg5^flox/flox^* mice were inoculated with R. australis i.v. at a dose of 3 × 10^5^ PFU per mouse. On day 4 p.i., the mice were euthanized. Mouse serum and tissues, including lung, liver, and spleen tissues, were collected. (A) The spleens of infected *Atg5^flox/flox^* Lyz-*Cre* and *Atg5^flox/flox^* mice were processed for immunohistochemical analysis of R. australis. Rickettsiae were stained red (as indicated by white arrows). (B) The rickettsial loads in mouse lung, liver, and spleen tissues were measured by quantitative real-time PCR. The number of rickettsial citrate synthase (CS) gene copies per nanogram of tissue genomic DNA represents the quantity of rickettsiae. (C) The serum levels of IL-1β and IL-6 in uninfected and infected mice were measured by Bio-Plex assay (Bio-Rad). Each mouse group included at least 3 to 5 mice. The data shown represent those from three independent experiments. *, *P* < 0.05; n.s., not significantly different.

*Atg5^flox/flox^* Lyz-*Cre* mice have been developed and employed in previous studies (32–34). Briefly, the *Atg5* gene was deleted from monocytes/macrophages and granulocytes by breeding *Atg5^flox/flox^* mice (33) to mice expressing the *Cre* recombinase from the endogenous lysozyme M locus to generate *Atg5^flox/flox^* Lyz-*Cre* mice. Deletion of the *Atg5* gene in these cells results in a deficit in autophagy (32–34). To determine the physiological importance of *Atg5* in R. australis infection *in vivo*, we challenged *Atg5^flox/flox^* Lyz-*Cre* and *Atg5^flox/flox^* mice with R. australis intravenously (i.v.). *Atg5^flox/flox^* Lyz-*Cre* mice were less supportive for the *in vivo* R. australis infection than *Atg5^flox/flox^* mice, as evidenced by lower rickettsial loads in tissues (Fig. 1A and B). Immunohistochemical staining with an antibody (Ab) directed against ATG5 confirmed the deficiency of ATG5 in host granulocytes/macrophages (see Fig. S1 in the supplemental material). As demonstrated in Fig. 1A, the quantity of R. australis in the spleens of infected mice was determined by immunohistochemical analysis using an Ab against rickettsiae. On day 4 postinfection (p.i.), the number of rickettsiae (stained in red and shown with white arrows in Fig. 1A) was dramatically greater in the spleens of *Atg5^flox/flox^* mice than in those of *Atg5^flox/flox^* Lyz-*Cre* mice. Consistent with these results, we found that the concentrations of R. australis in the liver, lung, and spleen of *Atg5^flox/flox^* mice were significantly greater than those in the organs of *Atg5^flox/flox^* Lyz-*Cre* mice by quantitative real-time PCR (Fig. 1B). The concentrations of R. australis in mice deficient in *Atg5* in macrophages were reduced approximately 90% in lung, 56% in liver, and 75% in spleen compared to those in the organs of *Atg5^flox/flox^* mice. One of the major characteristics of mouse models of rickettsial infection is the progression of the disease resulting from the progressively increased bacterial replication, measured by quantitative real-time PCR, in the various infected tissues *in vivo* (30, 35, 36). Although we did not show the concentrations of R. australis in tissues at time points earlier than day 4 p.i., the greater concentrations in lung, liver, and spleen in *Atg5*-competent mice than in those in *Atg5*-deficient mice most likely suggest that *Atg5* is required for the significant expansion of R. australis *in vivo*. Interestingly, a deficiency of *Atg5* in macrophages resulted in significantly enhanced *in vivo* levels of IL-1β in R. australis-infected mice compared to those in their *Atg5*-competent counterparts (Fig. 1C), suggesting that *Atg5* expression in macrophages negatively regulates the *in vivo* production of IL-1β during R. australis infection. As a control, we did not observe any significantly different levels of IL-6 production in the sera of infected *Atg5^flox/flox^* Lyz-*Cre* mice and *Atg5^flox/flox^* mice (Fig. 1C), suggesting that *Atg5* in macrophages specifically inhibits the levels of IL-1β production *in vivo* during R. australis infection. Thus, our results clearly demonstrate that *Atg5* expression in macrophages favors R. australis infection *in vivo*, which results in a greater bacterial load. Autophagy-related genes, including *Atg5*, have been reported to suppress proinflammatory cytokines in a variety of infection models (37–40). Thus, we hypothesized that *Atg5* in macrophages favors R. australis infection *in vivo* in association with the specific inhibition of the inflammatory cytokine IL-1β.

### Intracellular infection of R. australis in primary mouse macrophages *in vitro* is also *Atg5* dependent.

**FIG 2:**
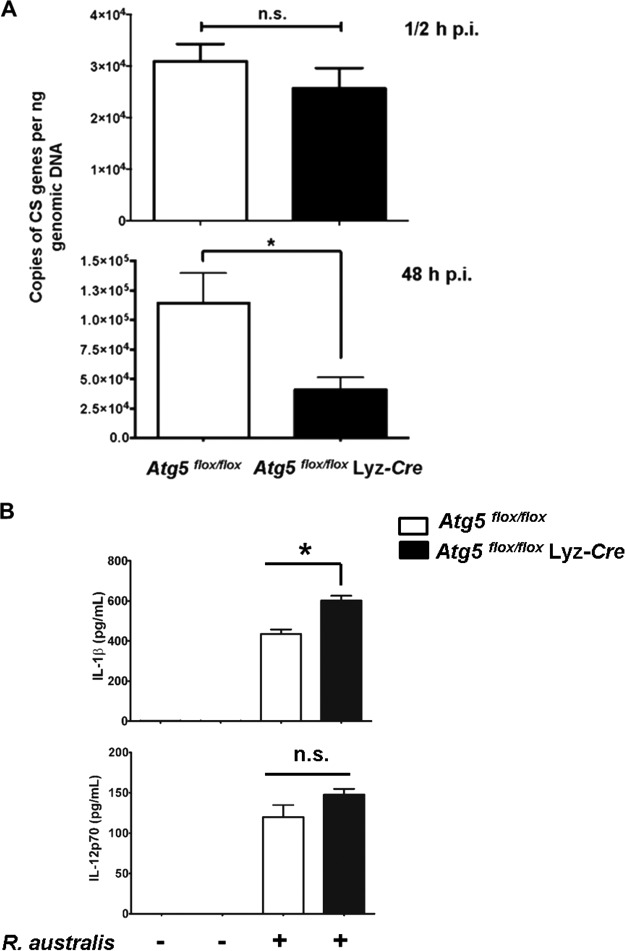
*Atg5* supports R. australis infection in BMMs accompanied by inhibited production of IL-1β. BMMs of *Atg5^flox/flox^* Lyz-*Cre* and *Atg5^flox/flox^* mice were infected with R. australis at an MOI of 2. (A) Rickettsial infection in these primary mouse macrophages was evaluated by quantitative real-time PCR at 30 min and 48 h p.i. The number of rickettsial citrate synthase (CS) gene copies per nanogram of genomic DNA represents the quantity of rickettsiae. (B) The production levels of IL-1β and IL-12p70 in the supernatant of R. australis-infected BMMs from *Atg5^flox/flox^* Lyz-*Cre* and *Atg5^flox/flox^* mice after 24 h were determined by Bio-Plex assay (Bio-Rad). The data shown are the mean ± standard deviation (SD) from three independent experiments. *, *P* < 0.05; n.s., not significantly different.

Next, we investigated whether *Atg5* in macrophages supports rickettsial infection *in vitro*. It is known that peritoneal macrophages and bone marrow-derived macrophages (BMMs) from *Atg5^flox/flox^* Lyz-*Cre* mice lack *Atg5* (41). We first determined whether *Atg5* is involved in the accumulation of R. australis in BMMs. As shown in Fig. 2A, at 30 min p.i., no significant difference in the rickettsial loads in the BMMs of *Atg5^flox/flox^* Lyz-*Cre* mice and *Atg5^flox/flox^* mice was detected (Fig. 2A). These results demonstrate that there is no significant difference in the initial concentrations of intracellular R. australis in the macrophages of *Atg5^flox/flox^* Lyz-*Cre* mice and those of *Atg5^flox/flox^* mice. Interestingly, at 48 h p.i., the concentrations of intracellular R. australis in the BMMs of *Atg5^flox/flox^* Lyz-*Cre* mice were significantly less (approximately 40%) than those in the BMMS of *Atg5^flox/flox^* mice (Fig. 2A). To determine whether the increased concentrations of R. australis in *Atg5^flox/flox^* BMMs compared with those in *Atg5*^flox/flox^ Lyz-*Cre* BMMs results from enhanced host cell viability, we examined the viability of BMMs from these transgenic mice at 48 h p.i. We did not observe significant cell death in either R. australis-infected *Atg5^flox/flox^* BMMs or infected *Atg5^flox/flox^* Lyz-*Cre* BMMs (Fig. S2) by flow cytometric analysis. Therefore, our *in vitro* results recapitulated our *in vivo* results by showing that *Atg5* favored intracellular R. australis infection in primary mouse macrophages.

Next, we determined the production levels of IL-1β and IL-12p70 by R. australis-infected BMMs of *Atg5^flox/flox^* and *Atg5^flox/flox^* Lyz-*Cre* mice after 24 h. As shown in Fig. 2B, IL-1β was produced at a significantly greater level by infected *Atg5^flox/flox^* Lyz-*Cre* BMMs than by *Atg5^flox/flox^* BMMs. No significant differences in the levels of IL-12p70 produced by infected BMMs of *Atg5^flox/flox^* Lyz-*Cre* mice and those produced by the BMMs of their *Atg5*-competent counterparts were observed. We have recently demonstrated that IL-1β production by R. australis-infected BMMs is mediated by a caspase-1-dependent inflammasome (31). Thus, these results suggest that *Atg5* deficiency enhances inflammasome-mediated IL-1β production by macrophages infected with R. australis.

### *Atg5* supports intracellular accumulation of R. australis in primary mouse macrophages in association with an inhibitory effect on rickettsiae mediated by IL-1β.

**FIG 3:**
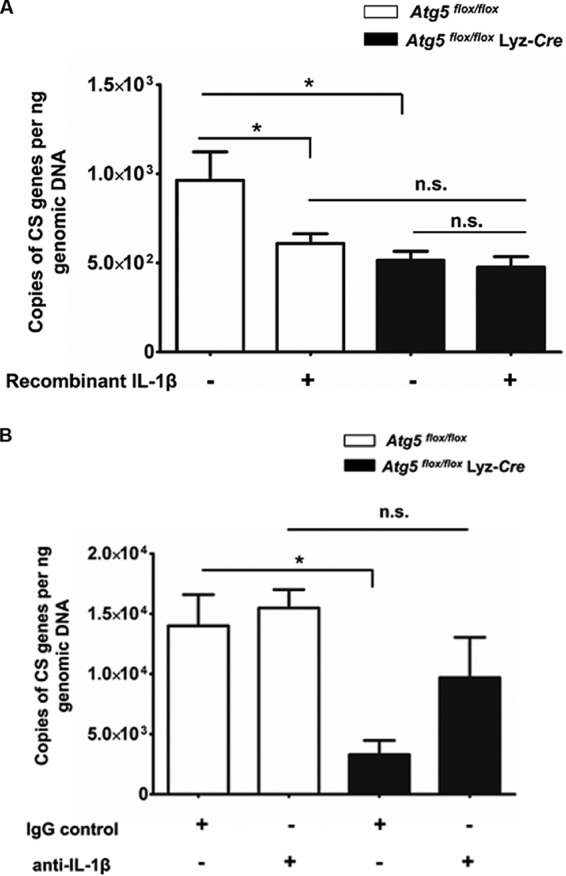
*Atg5* favors R. australis infection in BMMs in association with suppression of the IL-1β-mediated response. BMMs of *Atg5^flox/flox^* Lyz-*Cre* and *Atg5^flox/flox^* mice were infected with R. australis at an MOI of 2. (A) Simultaneously, these macrophages were treated with recombinant IL-1β. (B) Macrophages were simultaneously treated with neutralizing antibody against IL-1β and the IgG controls. At 48 h p.i., cells were washed and collected for quantitative analysis of intracellular R. australis by real-time PCR. The number of citrate synthase (CS) gene copies per nanogram of genomic DNA represents the quantity of rickettsiae. Each group included cells in 8 to 18 independent wells representing 2 to 3 mice. The data shown represent those from two independent experiments. *, *P* < 0.05; n.s., not significantly different.

Although the *in vivo* results that enhanced concentrations of R. australis in *Atg5*-competent macrophages are accompanied by suppressed IL-1β production were recapitulated by the *in vitro* data, it is still plausible that the *Atg5*-dependent accumulation of R. australis in primary mouse macrophages is associated with IL-1β. To address this question, we treated R. australis-infected *Atg5^flox/flox^* BMMs and *Atg5^flox/flox^* Lyz-*Cre* BMMs with exogenous recombinant IL-1β. At 48 h p.i., the concentrations of R. australis in *Atg5^flox/flox^* BMMs treated with recombinant IL-1β were significantly less than those in untreated cells (Fig. 3A). These results suggest that exogenous IL-1β plays a part in reducing the concentration of rickettsiae in *Atg5^flox/flox^* BMMs, mostly likely through inhibiting the accumulation of R. australis. Interestingly, the greater concentrations of R. australis in *Atg5^flox/flox^* BMMs than in *Atg5^flox/flox^* Lyz-*Cre* BMMs were abolished by treatment with IL-1β. However, the concentrations of R. australis in *Atg5^flox/flox^* Lyz-*Cre* BMMs were not altered upon treatment with recombinant IL-1β. It is possible that R. australis is not able to accumulate or grow in the absence of *Atg5* by an IL-1β-independent mechanism. To address this issue, we evaluated the rickettsial loads in cells treated with neutralizing antibodies against IL-1β and their corresponding IgG controls. Our results showed that *Atg5^flox/flox^* Lyz-*Cre* BMMs treated with neutralizing antibodies against IL-1β, but not those treated with the IgG controls, contained concentrations of intracellular R. australis comparable to those in *Atg5^flox/flox^* BMMs (Fig. 3B). The concentrations of intracellular R. australis in *Atg5*-deficient BMMs increased upon treatment with neutralizing antibodies against IL-1β compared with those in the IgG controls (Fig. 3B). These results suggest that R. australis is able to accumulate in the absence of *Atg5* in association with the IL-1β-mediated response. Neutralizing antibodies against IL-1β did not alter the concentrations of intracellular R. australis in *Atg5*-competent BMMs. Therefore, these results suggest that the increased *Atg5*-dependent accumulation of R. australis in primary mouse macrophages is closely associated with a reduced IL-1β-mediated inhibitory effect on rickettsiae.

### R. australis induces an autophagy-related response instead of active autophagy in *Atg5^flox/flox^* BMMs but not in *Atg5^flox/flox^* Lyz-*Cre* BMMs.

**FIG 4:**
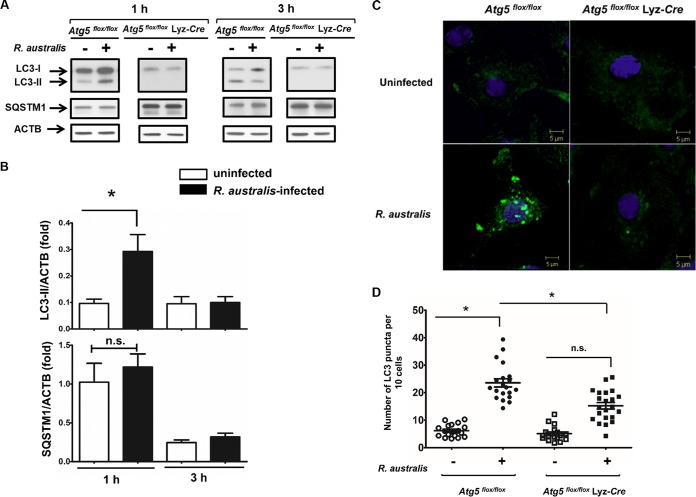
R. australis induces an autophagy-related response instead of active autophagy in *Atg5^flox/flox^* BMMs but not in *Atg5^flox/flox^* Lyz-*Cre* BMMs. BMMs were isolated from *Atg5^flox/flox^* Lyz-*Cre* and *Atg5^flox/flox^* mice and then infected with R. australis at an MOI of 5. (A) Induction of autophagosomes by rickettsiae in these infected macrophages was evaluated by determination of the expression levels of LC3-II and p62/SQSTM1 at different time points. The data shown are the mean ± standard deviation (SD) from three independent experiments. (B) The ratios of LC3-II/ACTB and SQSTM1/ACTB in uninfected and infected *Atg5^flox/flox^* BMMs were analyzed by densitometry. (C) Representative confocal microscopic images of infected BMMs of *Atg5^flox/flox^* and *Atg5^flox/flox^* Lyz-*Cre* mice at 1 h p.i. Green, LC3^+^ puncta; blue, DAPI (nuclei). (D) The average number of LC3 puncta per 10 cells was counted. More than 150 cells from 15 randomly selected images were counted. *, *P* < 0.05; n.s., not significantly different.

To further determine whether *Atg5*-promoted rickettsial infection is associated with autophagy, we examined autophagy flux in R. australis-infected BMMs from *Atg5^flox/flox^* Lyz-*Cre* and *Atg5^flox/flox^* mice. The conversion of LC3-I to lipidated LC3-II is a hallmark of autophagy and indicates autophagosome formation (42). The amount of LC3-II correlates directly with the number of autophagosomes. However, LC3 has also been shown to accumulate independently of autophagosomes (43). At 1 h p.i., but not 3 h p.i., R. australis induced a significantly increased level of LC3-II in *Atg5*-competent (*Atg5^flox/flox^*) macrophages compared to those in the uninfected controls (Fig. 4A and B). However, since LC3-II itself is degraded by autophagy, an increased number of autophagosomes may represent either induction of autophagy or blockade of any step downstream of autophagosome formation. To address this issue, we examined the levels of p62/SQSTM1, a ubiquitin-binding protein that is specifically degraded by autolysosomes. p62/SQSTM1 is considered a useful marker for autophagic vesicle turnover (44), as the level of p62/SQSTM1 is inversely correlated with autophagy flux. At both 1 h and 3 h p.i., R. australis did not induce a significantly reduced level of p62/SQSTM1 in *Atg5^flox/flox^* BMMs compared to that in the uninfected controls (Fig. 4A and B). These results suggest that R. australis induced a modified autophagy process without active autophagic vesicle turnover in *Atg5*-competent BMMs.

BMMs from *Atg5^flox/flox^* Lyz-*Cre* mice have been shown not to efficiently convert LC3-I to LC3-II (34). As expected, we did not observe any significant expression levels of lipidated LC3-II, but a dramatically increased amount of p62/SQSTM1 was observed in *Atg5^flox/flox^* Lyz-*Cre* macrophages compared to *Atg5^flox/flox^* macrophages (Fig. 4A). Further studies are required to demonstrate whether R. australis blocks the fusion of autophagosomes with lysosomes, resulting in an unaltered level of p62/SQSTM1.

We next evaluated whether autophagosomes are induced by R. australis in *Atg5*-competent and *Atg5*-deficient BMMs by examining LC3 puncta via confocal immunofluorescence microscopic analysis. We found LC3 puncta, labeled in green, in R. australis-infected *Atg5^flox/flox^* BMMs but not in *Atg5^flox/flox^* Lyz-*Cre* BMMs or in the uninfected controls (Fig. 4C). Quantitative analysis showed that the amount of LC3 puncta was significantly increased in *Atg5*-competent BMMs but not in *Atg5*-deficient BMMs compared to that in the uninfected controls (Fig. 4D). The amount of LC3 puncta in *Atg5^flox/flox^* BMMs was significantly greater than that in *Atg5^flox/flox^* Lyz-*Cre* BMMs. These results suggest that LC3 puncta in R. australis-infected BMMs accumulated via an *Atg5*-dependent mechanism.

### Ultrastructural analysis of R. australis-infected B6 BMMs.

**FIG 5:**
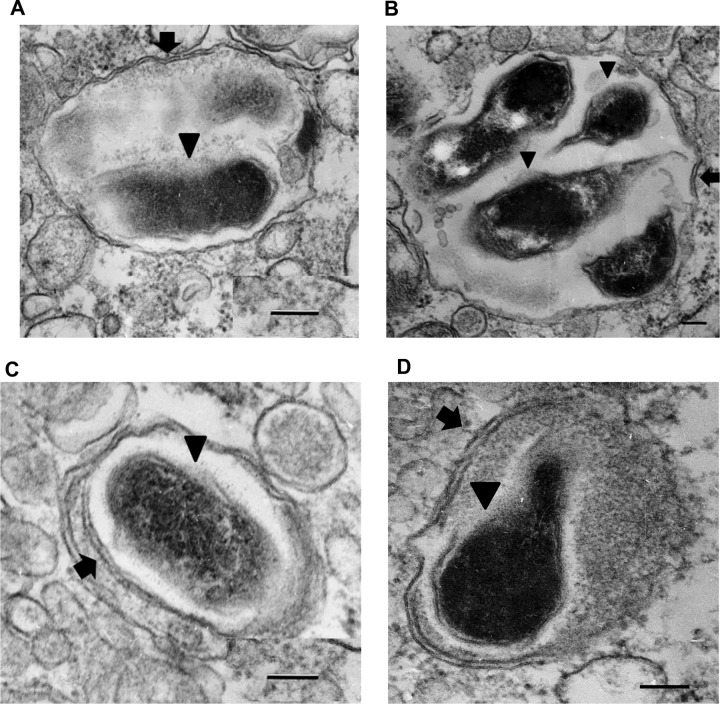
Transmission electron microscopy of R. australis-infected BMMs. Double-membrane-bound vacuoles containing rickettsiae (arrowheads) were found at 1 h (A, B) and 3 h (C, D) p.i. Arrow, double membranes either as fragments (A, B and D) or as a complete double membrane (C). Bars = 100 nm. Data represent those from three independent experiments.

Although we observed increased expression levels of LC3-II and LC3 puncta in *Atg5*-competent BMMs, it remained unclear if autophagosomes were accumulated by infection with R. australis. To this end, we studied the interplay of R. australis with autophagy in BMMs isolated from B6 mice at the ultrastructural level. Transmission electron microscopy is the most traditional method to study mammalian autophagy. An autophagosome is defined as a double-membrane-enclosed vacuole containing undigested cytoplasmic contents (42). At the ultrastructural level, in *R. australis*-infected BMMs, we regularly found individual intact rickettsiae or groups of rickettsiae in vacuoles surrounded by double membranes (indicated by arrows) at both 1 h p.i. (Fig. 5A and B) and 3 h p.i. (Fig. 5C and D) around at least a part of their circumference. Rickettsiae (indicated by arrowheads) were found inside these vacuoles (Fig. 5). These autophagosome-related vesicles were observed in multiple fields and nonserial sections but not in the negative controls. Some autophagosomes had only fragments of inner membrane (Fig. 5A, B, and D, arrows), suggesting that they were either gradually transforming into autophagolysosomes or generated due to a block that occurred after autophagosome formation.

### R. australis infection does not actively induce autophagic flux, although a small portion of cytosolic bacteria is colocalized with LC3 in BMMs from B6 mice.

**FIG 6:**
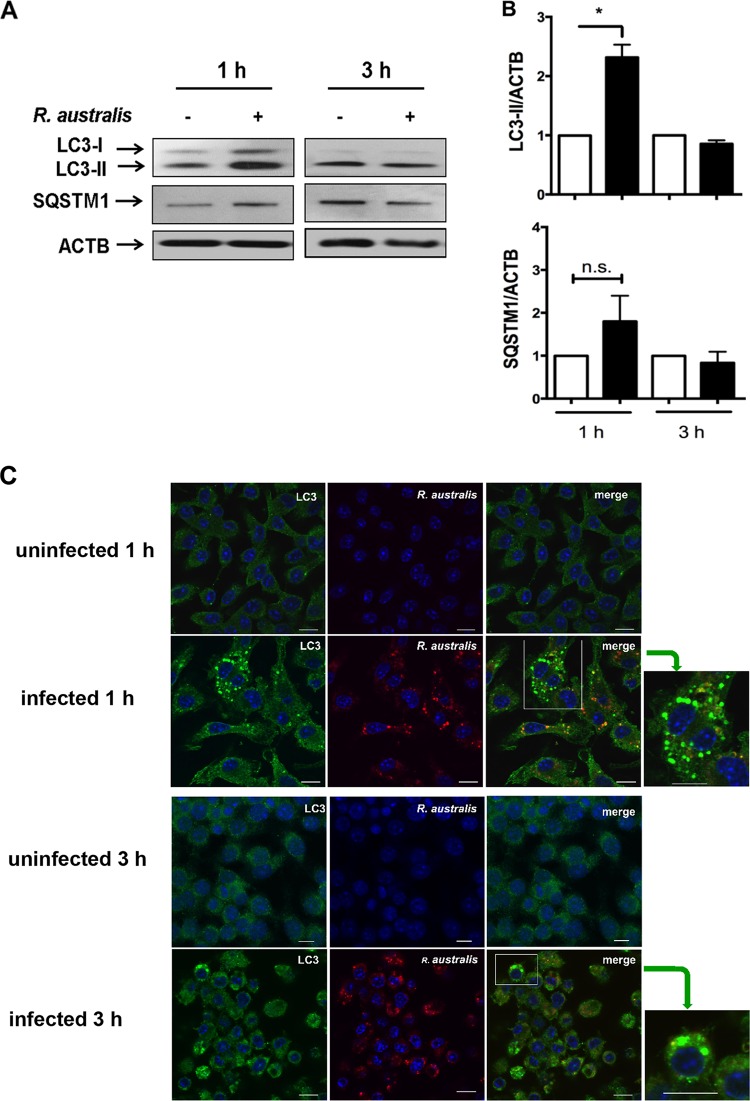

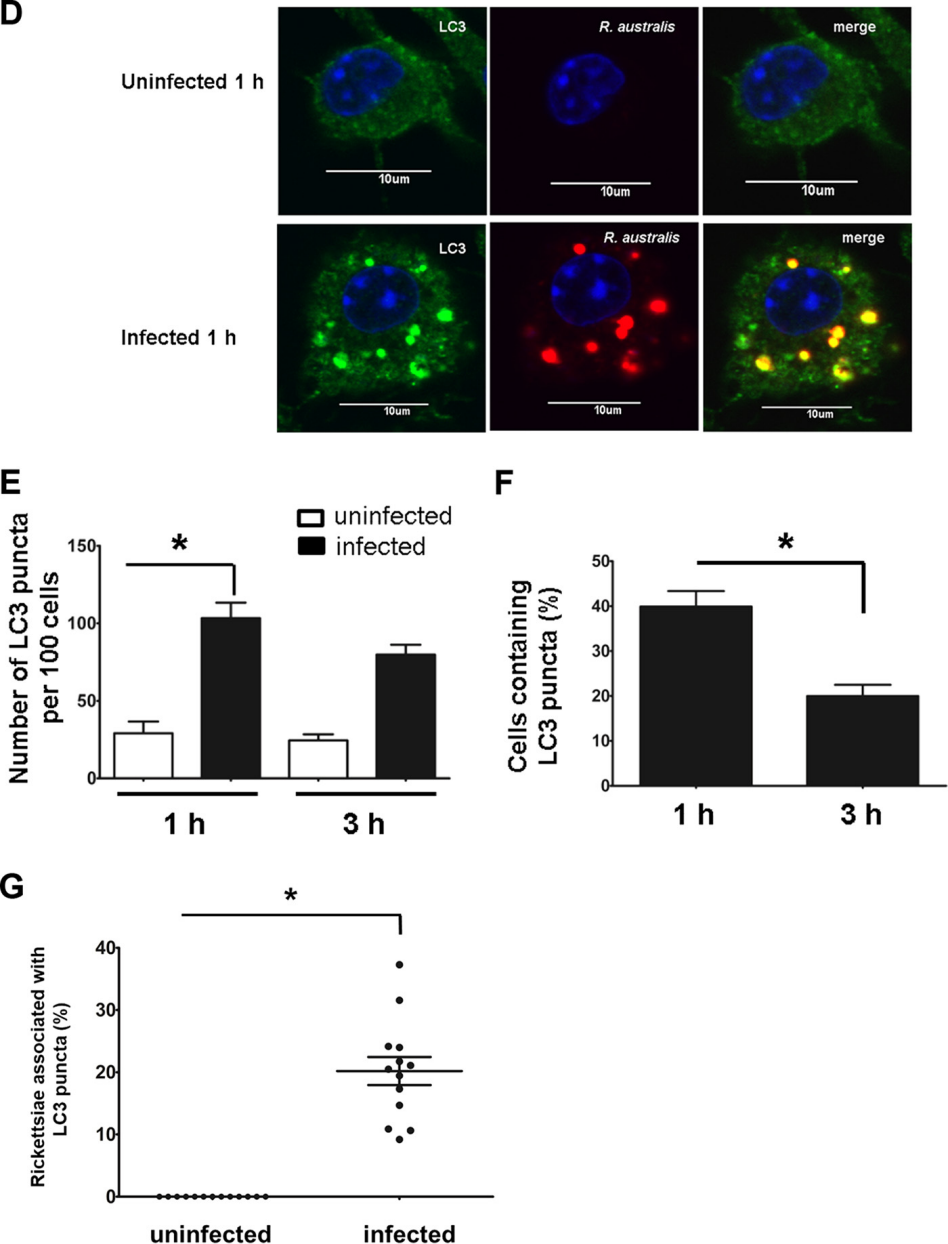
R. australis infection does not actively induce autophagic flux, although a small portion of cytosolic bacteria was colocalized with LC3 in BMMs from B6 mice. BMMs of WT B6 mice were infected with R. australis at an MOI of 5. Cells were collected at 1 h and 3 h p.i. (A) Cell lysates were immunoblotted with Abs directed against LC3 and p62/SQSTM1. (B) The ratios of LC3-II/ACTB and SQSTM1/ACTB were analyzed by densitometry. (C and D) Representative confocal microscopic images of uninfected and infected BMMs at 1 h and/or 3 h p.i. Bars = 10 μm. Green, LC3 puncta; blue, nuclei (DAPI); red, R. australis. (C) As indicated by the green arrows, the boxes on the right highlight representative cells containing LC3 puncta at a high magnification. (D) The colocalization of R. australis with LC3-positive compartments is depicted in yellow in the cytosol. (E to G) The percentage of cells containing LC3 puncta (E), the number of LC3 puncta per 100 cells (F), and the percentage of rickettsiae colocalized with LC3 (G) were determined. More than 300 cells from 12 randomly selected images were counted at each time point. The data shown are the mean ± standard deviation (SD) from two independent experiments. *, *P* < 0.05; n.s., not significantly different.

To further investigate the *Rickettsia*-associated autophagosome-like compartments observed by electron microscopic analysis in infected wild-type (WT) B6 BMMs, we examined the expression levels of LC3-II and p62/SQSTM1 by immunoblotting. At 1 h p.i., R. australis induced a significantly increased LC3-II/β-actin (ACTB) ratio accompanied by an unaltered SQSTM1/ACTB ratio compared to that for the uninfected controls (Fig. 6A and B). Thus, in line with the observations made above (Fig. 4A and B), our results suggest that R. australis fails to induce autophagy flux at the very early stage of infection, considering that the levels of p62/SQSTM1 did not reduce upon infection.

We next evaluated by confocal immunofluorescence microscopic analysis whether the autophagosome-like compartments are induced by R. australis in BMMs from WT mice. Our previous studies have shown a high infection rate in both R. conorii- and R. australis-infected mammalian host cells (31, 45). As shown in Fig. 6C, R. australis (red) was detected in the cytosol (nucleus as blue) of most (more than 90%) of the infected BMMs, whereas it was not found in the uninfected controls, suggesting that these bacteria showed a substantial infectivity rate in primary murine macrophages. In line with the results obtained by immunoblotting, confocal immunofluorescence microscopic analysis demonstrated LC3-positive organelles or LC3 puncta (green) in R. australis-infected BMMs at both 1 h and 3 h p.i. (Fig. 6C). To further determine the association of these LC3-positive organelles with R. australis, we examined their colocalization with R. australis. Some of these LC3-positive organelles, labeled green, colocalized with R. australis in the cytosol, shown in yellow (Fig. 6C and D). Quantitatively, the LC3 puncta were numerous and significantly increased at 1 h p.i. compared to their levels in the uninfected controls (Fig. 6C and E). At 3 h p.i., the quantity of LC3 puncta was substantial but not significantly different from that in the uninfected controls (Fig. 6E). About 36.9% ± 9.9% of infected cells contained LC3 puncta at 1 h p.i., and their levels were significantly decreased at 3 h p.i. (Fig. 6F). Further quantitative analysis revealed that 20.1% ± 8.1% of rickettsiae colocalized with autophagosomes at 1 h p.i. (Fig. 6G). These results suggest that autophagy-related compartments, presumably autophagosomes, accumulated at the very early stage of infection. Only a small proportion of rickettsiae were associated with these autophagosomes, although almost all of the cells were infected with rickettsiae. It remains unknown why only part of the infected cells contained LC3 puncta and why a small portion of rickettsiae were colocalized with an LC3-positive compartment.

### Treatment with rapamycin significantly facilitates R. australis infection in BMMs.

**FIG 7:**
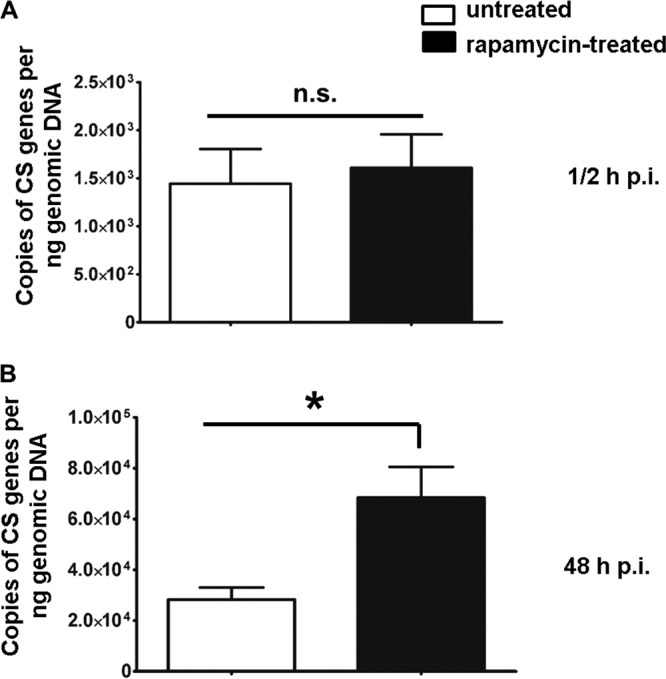
Autophagy, induced by an mTOR inhibitor, significantly promotes the infection of R. australis in BMMs. WT B6 BMMs were pretreated with rapamycin (50 nM) for 4 h and then infected with R. australis at an MOI of 2. At 30 min (A) and 48 h (B) p.i., the adherent cells were collected for quantification of intracellular bacteria after the cells were washed with PBS. Infection of R. australis in these treated and untreated BMMs was evaluated by quantitative real-time PCR. The number of rickettsial citrate synthase (CS) gene copies per nanogram of genomic DNA represents the concentration of rickettsiae. Each group included cells in at least 10 independent wells in each experiment. The data shown are the mean ± standard deviation (SD) from two independent experiments. *, *P* < 0.01; n.s., not significantly different.

Although our results have shown that *Atg5* supports R. australis infection in association with autophagosomes in genetically modified BMMs, it remains incompletely clear whether autophagy facilitates R. australis infection. To answer this question, we examined the concentrations of rickettsiae in BMMs treated with rapamycin, a classic autophagy inducer. Autophagy is negatively regulated by the mammalian target of rapamycin (mTOR) and can be induced in all mammalian cell types by the mTOR inhibitor rapamycin (46). WT BMMs were pretreated with a low concentration of rapamycin and then infected with R. australis. We did not find any significant cell death in BMMs treated with rapamycin. At 30 min p.i., we did not find any significant difference in the concentrations of intracellular R. australis in rapamycin-treated BMMs from those in untreated cells (Fig. 7A). These results suggest that autophagy induction by rapamycin does not have an impact on the concentrations of intracellular rickettsiae at the beginning of the infection. However, at 48 h p.i., the concentrations of intracellular R. australis in rapamycin-treated BMMs were significantly greater than those in the untreated controls (Fig. 7B). These results suggest that autophagy induction promotes R. australis infection, in line with our results obtained using BMMs from *Atg5*-conditional-knockout mice (Fig. 2A). These data indirectly suggest that the enhanced concentrations of intracellular *R. australis* in *Atg5*-competent BMMs are associated with a host autophagic response, although further investigations are required to reveal the mechanisms involved.

## DISCUSSION

These studies demonstrate for the first time that one of the autophagy-related genes, *Atg5*, favors R. australis infection in macrophages. More strikingly, *Atg5* expression in macrophages contributes to the pathogenesis of rickettsial diseases*. Atg5* supports the accumulation of R. australis in macrophages in association with downregulation of the inhibitory effect on rickettsiae mediated by the proinflammatory cytokine IL-1β. R. australis promotes the accumulation of *Atg5*-dependent LC3 but does not actively induce an autophagic flux in primary mouse macrophages at the very early stage of infection. Furthermore, our data suggest that exogenous induction of autophagy facilitates R. australis infection in macrophages, as evidenced by significantly increased concentrations of intracellular rickettsiae in response to treatment with rapamycin. Although it is not completely clear how R. australis exploits *Atg5*-mediated mechanisms to support bacterial infection in macrophages, our studies reveal that *Atg5*, a gene with a previously unknown function in rickettsial infection, could potentially serve as a target for the design of therapeutic strategies for rickettsioses in the future.

Both human and animal studies have demonstrated that macrophages are critically involved in the initial establishment of rickettsial infections (7–10). It is still poorly understood how rickettsiae modulate macrophage behavior to facilitate the progression of the diseases. Our studies employed conditional-knockout mice to investigate cell-specific mechanisms mediating the pathogenesis of rickettsial disease. Although macrophages have been shown to be of principal interest in these conditional-knockout mice, *Atg5*-deficient Lyz-expressing cells other than macrophages, such as neutrophils, are also possibly involved in controlling rickettsial infection in *Atg5^flox/flox^* Lyz-*Cre* mice *in vivo*. Our results, more strikingly, recapitulated the *in vitro Atg5*-dependent infection by R. australis in macrophages in a mouse model of Rocky Mountain spotted fever (28). Therefore, it is most likely that macrophages are the responsible cells mediating the difference in rickettsial bacterial burdens *in vivo* in these conditional-knockout mice. Since rickettsiae primarily target microvascular endothelial cells, future investigations are required to identify whether changes in rickettsial concentration in tissues are due only to cell-intrinsic effects of the *Atg5* gene on rickettsial infection in macrophages and/or to the effects of *Atg5* genes acting in *trans* from macrophages to endothelial cells.

Our findings illustrate that R. australis exploits *Atg5*-mediated mechanisms to support bacterial infection in association with dampening of the IL-1β-mediated antirickettsial effect in macrophages. IL-1β-mediated bacterial killing has been demonstrated in infections of macrophages with other extracellular bacteria, such as Pseudomonas aeruginosa (47). Although the concentrations of intracellular R. australis were significantly reduced in response to treatment with IL-1β in *Atg5*-competent macrophages, it is unclear whether IL-1β is capable of exerting rickettsial killing activity. Future studies quantifying live R. australis bacteria in *Atg5^flox/flox^* Lyz-*Cre* BMMs versus *Atg5^flox/flox^* BMMs at early and later time points will further reveal whether *Atg5* supports the replication or survival of R. australis in macrophages. Interestingly, treatment with exogenous IL-1β failed to inhibit the accumulation of R. australis in *Atg5^flox/flox^* Lyz-*Cre* BMMs (Fig. 3). Why? We propose that *Atg5^flox/flox^* Lyz-*Cre* BMMs are fully activated by a large amount of IL-1β produced upon R. australis infection in an autocrine manner, which may result in inhibition of the intracellular growth of R. australis. Thus, *Atg5^flox/flox^* Lyz-*Cre* BMMs were unable to respond to stimulation with exogenous IL-1β due to the full autocrine activation. In contrast, infected *Atg5^flox/flox^* BMMs produced a minimal level of IL-1β, which may not be sufficient for autocrine activation of macrophages. In response to exogenous recombinant IL-1β, *Atg5^flox/flox^* BMMs exhibited an antirickettsial effect leading to a reduced concentration of intracellular R. australis. More strikingly, the greater rickettsial loads in *Atg5^flox/flox^* versus *Atg5^flox/flox^* Lyz-*Cre* BMMs were abrogated by treatment with exogenous recombinant IL-1β. These results suggest that inhibition of the IL-1β-mediated antirickettsial effect is associated with the *Atg5*-dependent accumulation of R. australis in primary mouse macrophages. Interestingly, no difference in the intracellular concentrations of R. australis was observed in *Atg5^flox/flox^* BMMs upon treatment with antibodies against IL-1β. These observations could be explained by the possibility that there was a minimal amount of IL-1β present to be neutralized by the antibodies. In addition, a deficiency in *Atg5* in infected macrophages enhanced the production of IL-1β by about 20% compared to the amount produced by infected *Atg5*-competent macrophages (Fig. 2B). However, addition of IL-1β reduced the concentrations of intracellular rickettsiae in *Atg5^flox/flox^* BMMs by nearly 50% (Fig. 3). These results suggest that effectors other than IL-1β may also contribute to controlling intracellular R. australis in macrophages. Therefore, our data suggest that *Atg5* contributes to the intracellular infection of R. australis in primary mouse macrophages in close association with downregulation of the inhibitory effect on rickettsiae mediated by autocrine-secreted IL-1β.

Numerous studies have identified *Atg* genes in macrophages to be key regulators of inflammation (37, 38, 48). *Atg* genes in macrophages are known to limit innate immune inflammation. In contrast, induced autophagy is also reported to augment IL-1β secretion (49). In the present study, deletion of the *Atg5* gene in macrophages resulted in elevated levels of the proinflammatory cytokine IL-1β but not those of IL-6 during rickettsial infection (Fig. 1C). Our recent studies have demonstrated that R. australis activates the caspase-1-dependent inflammasome, which mediates the production of biologically functional IL-1β by macrophages (31). Therefore, the significantly enhanced levels of production of IL-1β by *Atg5^flox/flox^* Lyz-*Cre* BMMs suggest that *Atg5* possibly plays a negative role in the activation of the inflammasome but not IL-6-mediated inflammation during rickettsial infection. It remains unknown whether the autophagosome is involved in inhibiting the inflammasome pathway during R. australis infection. Recent studies have demonstrated a general role of TRIMs as autophagic receptor regulators in regulating the immune response, with MEFV/TRIM20 specializing in the suppression of inflammasome and caspase-1 activation, leading to IL-1β production (50). As a cytosolic multiprotein complex, inflammasomes specifically detect danger signals and infectious pathogens in the cytosol. As bacteria residing free in the cytosol, a portion of the R. australis bacteria colocalized with autophagosomes at 1 h p.i. but did not induce active autophagy flux. These results lead us to the hypothesis that *Atg5*-dependent autophagosomes provide a shelter for these cytosolic bacteria, preventing them from being recognized by inflammasomes. Furthermore, the *Atg5*-dependent, autophagy-independent regulation of inflammation has also been reported (41). Thus, our results do not exclude the possibility that *Atg5* also favors R. australis infection in macrophages through an autophagy-independent mechanism.

It is not surprising to us that *R. australis*, a bacterium that appears to be ideally adapted to live in the cell cytoplasm, does not induce active autophagy but modifies the autophagic pathway. Listeria monocytogenes is not normally a target for autophagy and is taken into the autophagic pathway only when bacterial protein synthesis is inhibited (51). It is presumed that the very small percentage of dead R. australis bacteria generated in the process of purification is associated with the induction of autophagosomes. Future studies using R. australis bacteria treated with antibiotics, such as doxycycline or chloramphenicol, will help us to reveal the role of *de novo* protein synthesis by rickettsiae in the development of bacterium-associated autophagosomes. In addition, further investigations should focus on whether the autophagosomes observed in R. australis-infected BMMs result from blockage of the maturation of autolysosomes, which will lead to a better understanding of the mechanisms by which rickettsiae subvert the active autophagic response.

The interplay of R. australis with autophagosomes is distinct from what is seen in other genetically related intracellular bacteria. Rickettsia australis accumulated autophagosomes as early as 1 h p.i., which were then evaded by rickettsiae at 3 h p.i. Thus, our current results suggest that the accumulation of autophagosomes by R. australis occurred as a rapid, transient, and dynamic response. Group A *Streptococcus* (GAS) begins to colocalize with green fluorescent protein-LC3 at 3 h p.i. (52). Coxiella burnetii inclusions accumulate LC3 at 5 min p.i. (53). Orientia tsutsugamushi induces LC3 puncta at 2 h p.i. but actively escapes from autophagic recognition so that inhibition or activation of autophagy does not affect its intracellular growth (54, 55). Anaplasma phagocytophilum colocalizes with LC3 and Beclin 1 at 32 and 48 h p.i., respectively (22). Ehrlichia chaffeensis induces autophagy at 3 days p.i. (56, 57). C. burnetii recruits LC3 to the parasitophorous vacuole (PV), within which C. burnetii uniquely replicates (58). However, no change in the levels of p62/SQSTM1 is observed in C. burnetii-infected macrophages (58), which is similar to what we observed in R. australis-infected macrophages (Fig. 1 and 4). Winchell et al. recently demonstrated that C. burnetii exploits the p62/SQSTM1-regulated host signaling pathway for intramacrophage growth (59). Our study clearly indicated that macrophages did not control R. australis infection but, instead, supported its accumulation in an *Atg5*-dependent fashion. These results were further confirmed by the greater accumulation of R. australis in macrophages upon stimulation with the classic autophagy inducer, rapamycin. To further determine whether the phenotype that we observed is only due to *Atg5*, we compared the rickettsial concentrations in BMMs of *Atg16l1^flox/flox^* and *Atg16l1^flox/flox^*-LysM*Cre* mice. Similar to our results in *Atg5*-deficient macrophages, deletion of *Atg16l1* significantly reduced the concentrations of R. australis (see Fig. S3 in the supplemental material). Thus, our results clearly demonstrated a role of autophagy-related genes in supporting the infection of R. australis in macrophages. In our previous studies, R. conorii has been reported to be engulfed in autophagosomes and destroyed in structures resembling autolysosomes (60, 61). These results were obtained in mouse microvascular endothelial cells treated with cytokines, including gamma interferon and tumor necrosis factor alpha, which are known to be major immune effectors with rickettsicidal effects (62). Compared to the previous studies, the present studies greatly helped us with understanding the pathogenic mechanisms *in vivo*, before adaptive immunity is initiated, by focusing on the initial target cells of rickettsiae and on a state without external stimuli.

In conclusion, by employing conditional, autophagy-specific gene-knockout mice, our studies clearly indicated that macrophages play a critical role in the *in vivo* pathogenesis of rickettsioses via autophagy gene-mediated mechanisms. R. australis manipulates macrophages for the benefit of infection in association with dampening the host inflammatory response and modifying the autophagic pathway. Our findings provide novel insights into potential host-based therapeutic interventions against rickettsioses by targeting the autophagy gene-dependent pathways in myeloid cells.

## MATERIALS AND METHODS

### Rickettsiae and mice.

For *in vitro* infection, R. australis (Cutlack strain) was cultivated in Vero cells and purified by either Renografin density gradient centrifugation or the use of a Renografin cushion, as previously described (35, 63, 64). For mouse inoculation, R. australis was propagated in the yolk sacs of specific-pathogen-free embryonated chicken eggs as described previously (30). The concentrations of stock rickettsiae cultured in both yolk sacs and cell cultures were determined by plaque assay, as described in previous studies (30). The rickettsial stock was stored at −80°C until used, and all the experiments described in this study were performed in a certified biosafety level 3 (BSL3) laboratory at UTMB.

WT B6 mice were purchased from The Jackson Laboratory (catalog number 000664). *Atg5^flox/flox^* Lyz-*Cre* and *Atg5^flox/flox^* (control) mice were kindly provided by Noboru Mizushima at the Tokyo Medical and Dental University in Japan and Herbert Virgin IV at the Washington University School of Medicine in St. Louis, MO (33, 41). For *in vivo* experiments, *Atg5^flox/flox^* Lyz-*Cre* and *Atg5^flox/flox^* mice were inoculated i.v. through the tail vein with R. australis at a dose of 3 × 10^5^ PFU per mouse, which is a sublethal dose for WT B6 mice. Uninfected mice served as negative controls. After infection, the mice were monitored daily for signs of illness. On day 4 p.i., the mice were euthanized. Mouse tissues, including lung, liver, and spleen tissues, were collected for measurement of bacterial replication and histopathological and immunohistochemical analyses. All mice were maintained and manipulated in an animal biosafety level 3 (ABSL3) facility at UTMB. All experiments and procedures were approved by the UTMB Animal Care and Use Committee, and experiments in mice were performed according to the guidelines of the *Guide for the Care and Use of Laboratory Animals* (65).

### Generation of bone marrow-derived macrophages.

The generation of primary BMMs from 6- to 8-week-old female WT B6 mice, *Atg5^flox/flox^* mice, and *Atg5^flox/flox^* Lyz-*Cre* mice was performed as previously described (66). Briefly, after the femurs and tibias were dissected, the bone marrow was flushed and cells were cultivated in low-endotoxin Dulbecco modified Eagle medium containing 10% (vol/vol) newborn calf serum (catalog number 16010142; Gibco, Thermo Fisher Scientific) supplemented with either 20% supernatant from an L929 cell culture or a CMG14-12 cell culture or recombinant macrophage colony-stimulating factor (catalog number 315-02; PeproTech) at 37°C in 5% CO_2_ (67). On day 6 of culture, cells were harvested and characterized by flow cytometric analysis after staining with anti-F4/80 and CD11b Abs (catalog numbers 565410 and 553310; BD Bioscience). Approximately 90% of these cells were F4/80^+^ and CD11b^+^. These cells were plated in 24-well plates at a density of 1 × 10^6^ cells/well and used for the experiments within 24 h.

### *In vitro* infections of macrophages.

BMMs were infected with R. australis at a multiplicity of infection (MOI) of 2:1 or 5:1 as indicated below and in the figure legends. To synchronize bacterial internalization, rickettsiae were centrifuged onto the cells at 560 × *g* for 5 min. Cells were continuously incubated at 37°C in 5% CO_2_. At 1 h, 3 h, and 24 h p.i., cells were collected and washed for further experiments. Uninfected macrophages served as negative controls. Cells treated with Hanks’ balanced salt solution (HBSS; catalog number 14170112; Gibco, Thermo Fisher Scientific) for 4 h served as the positive controls of autophagy induction.

### Immunofluorescence microscopy.

For immunofluorescence detection of LC3 puncta and localization of LC3 and rickettsiae, cells were seeded on glass coverslips in 12-well plates 1 day before infection. At the time points indicated below, cells were washed with phosphate-buffered saline (PBS), fixed with 4% paraformaldehyde in PBS for 20 min, permeabilized with 0.5% Triton-X in PBS for 20 min, and blocked with 3% bovine serum albumin in PBS for 30 min. Samples were incubated with rabbit polyclonal Abs directed against R. australis and goat anti-mouse LC3 (catalog number sc-16755; Santa Cruz), followed by appropriate secondary Abs, including Alexa Fluor 488-conjugated chicken anti-goat IgG and Alexa Fluor 647-conjugated donkey anti-rabbit IgG (catalog numbers A21467 and A31573; Life Technologies). The anti-LC3 Ab preferentially labels autophagosome-associated LC3-II. Nuclei were stained with DAPI (4′,6-diamidino-2-phenylindole) in ProLong Gold antifade mountant (catalog number P-36931; Life Technology). Coverslips were sealed with nail polish and visualized by confocal microscopy (Olympus Fluoview 1000 microscope) using FV10-ASW software (Olympus). The percentage of cells containing LC3-positive (LC3^+^) puncta and the percentage of rickettsiae colocalized with LC3^+^ puncta in more than 300 cells or 300 bacteria from at least 12 randomly selected images were calculated using MetaMorph software and/or ImageJ software as previously described (68, 69).

### Transmission electron microscopy.

For examination of infected BMMs by electron microscopy, BMMs were harvested at the time points p.i. indicated above and immersed in Ito’s fixative (2.5% formaldehyde, 0.1% glutaraldehyde, 0.03% CaCl_2_, and 0.03% trinitrophenol in 0.05 M cacodylate buffer, pH 7.3) at room temperature for 1 h and then overnight at 4°C. After washing, samples were processed further as described previously (45). Ultrathin sections were cut on a Leica EM UC7 ultramicrotome (Leica Microsystems, Inc.) and examined in a Phillips 201 transmission electron microscope (Phillips Electron Optics) at 60 kV.

### Western immunoblotting.

For assessment of the cellular LC3 levels and the conversion of naive LC3-I to lipidated LC3-II, cells were lysed with radioimmunoprecipitation assay lysis buffer (catalog number 20-188; EMD Millipore) supplemented with protease inhibitors (catalog number 05892970001; Roche). The soluble part of the cell lysates was isolated by centrifugation and separated by SDS-PAGE, transferred to a polyvinylidene difluoride membrane, and probed with a rabbit polyclonal Ab directed against LC3B (catalog number 4108; Cell Signaling Technology). Immunoreactive bands were visualized using an appropriate secondary Ab and enhanced chemiluminescence detection reagents (catalog number 32106; Thermo Scientific, Pierce). Equal protein loading of the gels was monitored by detecting β-actin with mouse monoclonal antibody (catalog number A1978; Sigma, St. Louis, MO) in the cell lysates. The detection of p62/SQSTM1 is indicative of the autophagic process, as described previously (70). SQSTM1-specific immunoblotting was performed as described above using Abs directed against p62/SQSTM1 (catalog number 5114; Cell Signaling Technology). The amounts of LC3-II and p62/SQSTM1 relative to the amount of ACTB and the ratio of LC3-II to p62/SQSTM1 were calculated after densitometry measurements were made using ImageJ software or Image Studio Lite software (LI-COR Biosciences, Lincoln, NE) (71, 72).

### Induction of autophagy with rapamycin treatment.

For the study of the effect of autophagy on inhibiting or supporting bacterial infection, BMMs were incubated with medium containing rapamycin (catalog number R8781; Sigma, St. Louis, MO) at a concentration as low as 50 nM. The culture medium in each well was replaced with fresh medium containing rapamycin. After 4 h, the cells were infected with R. australis at an MOI of 2. At 1/2 h and 48 h p.i., BMMs were washed with PBS to remove extracellular bacteria, and intracellular bacterial replication was monitored using real-time PCR as described below. The activation effect of rapamycin on autophagy was examined by immunoblotting with antibodies against LC3 and p62/SQSTM1. Cell morphology was observed by microscopy, and cell viability was determined with the trypan blue dye exclusion method.

### Quantification of bacterial loads by quantitative real-time PCR.

To determine the number of intracellular rickettsiae following *in vitro* macrophage infection and *in vivo* mouse infection, R. australis-infected BMMs and mouse tissues, including lung, liver, and spleen tissues, were collected at the time points indicated above. DNA was extracted from these cells and tissues using a Qiagen DNA extraction kit (catalog number 69506; Valencia) as described previously (45). Quantitative real-time PCR was performed using an iCycler instrument from Bio-Rad (Hercules). Rickettsial loads were determined by real-time PCR with primers and TaqMan probes for the *Rickettsia*-specific citrate synthase (CS) gene (*gltA*) as described in our previous studies (*gltA* forward, GAGAGAAAATTATATCCAAATGTTGAT; *gltA* reverse, AGGGTCTTCGTGCATTTCTT; *gltA* probe, CATTGTGCCATCCAGCCTACGGT) (30). The *gltA* probe was labeled with 6-carboxyfluorescein (Biosearch Technologies). Two-step cycle parameters (95°C and 60°C) were used. The results were normalized to the number of nanograms of genomic DNA in the same sample and expressed as the CS gene copy number per nanogram of genomic DNA.

### Evaluation of *in vitro* and *in vivo* release of cytokines.

At 24 h p.i., the cell culture supernatant of infected BMMs of *Atg5^flox/flox^* mice and *Atg5^flox/flox^* Lyz-*Cre* mice was collected for assessment of the *in vitro* release of IL-1β and IL-12p70. The supernatant from uninfected BMMs served as a control. On day 4 p.i., serum samples were collected from infected *Atg5^flox/flox^* mice and *Atg5^flox/flox^* Lyz-*Cre* mice for evaluation of the levels of IL-1β and IL-6. Uninfected mouse serum served as a control. The analysis of these cytokines was performed on supernatant and serum samples using a magnetic bead-based multiplex immunoassay (Bio-Plex; Bio-Rad Laboratories) following the manufacturer’s instructions. The plates were read at 450 nm, and the absorbances were transformed to the number of picograms per milliliter using calibration curves prepared with cytokine standards included in the kit.

### Immunohistochemical analysis.

After fixation, tissues were embedded in paraffin, sectioned, and stained for immunohistochemical analysis. Antigen unmasking was performed by treatment with sodium citrate buffer at pH 6, and tissue sections were stained for R. australis using rabbit polyclonal Abs against rickettsiae and ATG5 (catalog number NB110-53818; Novus). Biotinylated secondary Abs (Vector Labs) were diluted in Dako Ab diluent (catalog number S3022; Dako). The tertiary reagents and streptavidin-alkaline phosphatase were diluted in the same Dako diluent, as were the primary Abs (73). All washes were with Tris-buffered saline–Tween 20 (Sigma). Sections were dehydrated before synthetic glass coverslips were mounted with Permount mounting medium. All the sections were photographed with an Olympus DP71 camera (Olympus, Center Valley, PA, USA) attached to an Olympus Ix71 inverted microscope (Olympus, Tokyo, Japan) utilizing ×20 objectives.

### Treatment with recombinant IL-1β or neutralizing antibodies against IL-1β.

For the study of how *Atg5*-dependent autophagy favors rickettsial replication, BMMs of *Atg5^flox/flox^* and *Atg5^flox/flox^* Lyz-*Cre* mice were infected with R. australis at an MOI of 2, as described above. At the time of infection, cells were incubated with medium containing either 10 ng/ml recombinant IL-1β (catalog number 401-ML; R&D Systems) or neutralizing Abs against IL-1β (catalog number 503504; BioLegend). Untreated cells and cells treated with the IgG isotype (catalog number 400902; BioLegend) were included as controls. The culture medium in each well was replaced with fresh medium every day to avoid starvation. The cell number was counted, the cell morphology was observed by microscopy, and cell viability was determined by the trypan blue dye exclusion method. At 48 h p.i., cells were collected, washed, and processed for quantitative analysis of rickettsiae using real-time PCR, as described above.

### Statistical analysis.

For comparison of multiple experimental groups, the one-way analysis of variance (ANOVA) with Bonferroni’s correction procedure was used. Two-group comparisons were conducted using either Student’s *t* test or Welch’s *t* test, depending on whether the variance between two groups was significantly different. When two factors were included in the comparison, two-way ANOVA with the Bonferroni posttest was used. All the statistical analyses were performed using GraphPad Prism software (version 5.01). *P* values of 0.05 or less were the threshold for statistical significance.

## Supplementary Material

Supplemental file 1
