# Supplementary material for: Atg5 Supports Rickettsia australis Infection in Macrophages In Vitro and In Vivo
Source: Infect Immun. 2018 Dec 19;87(1):e00651-18. doi: 10.1128/IAI.00651-18 (PMC6300621; doi:10.1128/IAI.00651-18)
Supplement: Supplemental file 1 [file 786f5461def39be8fc9282987bd4f53a_IAI.00651-18-s0001.pdf]

Supplementary FIG 1

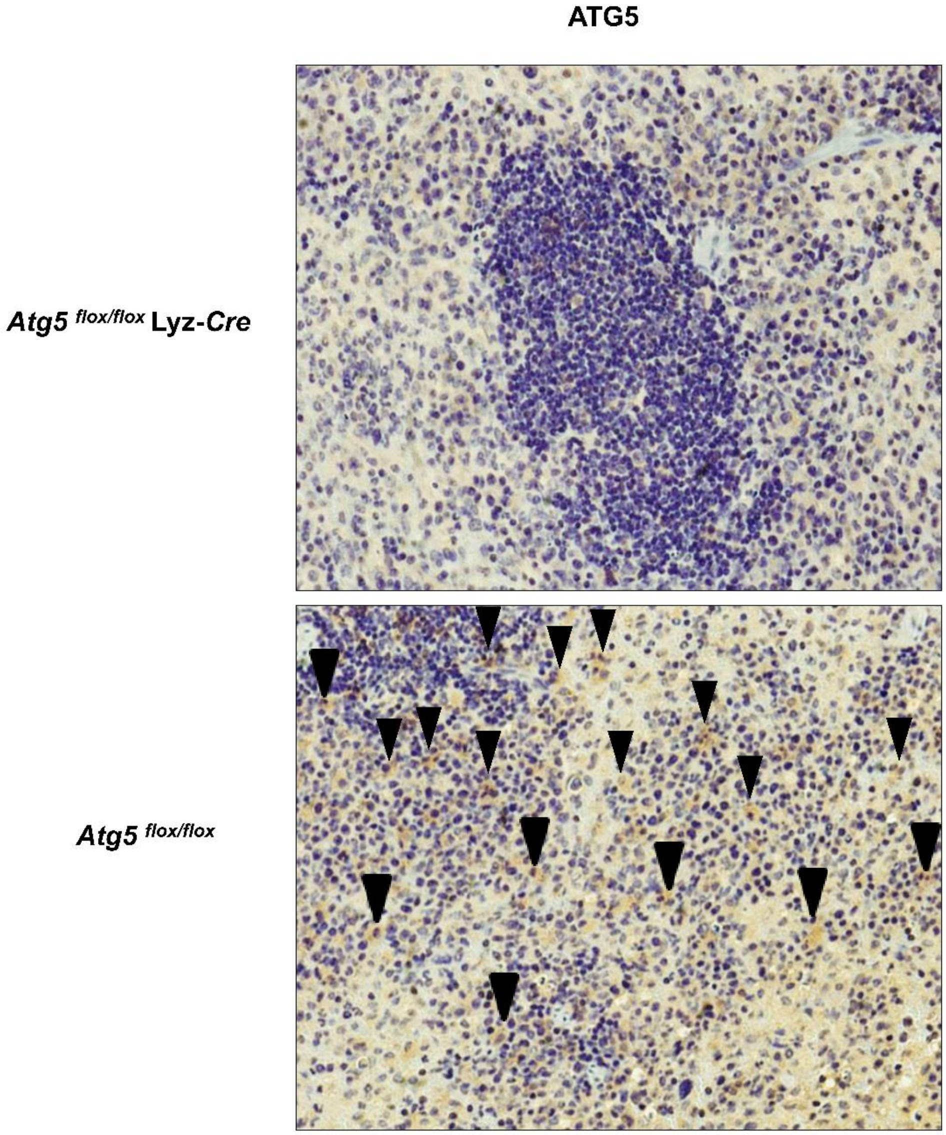

Supplementary FIG 1.Immunohistochemical staining of ATG5 in *Atg5* $^{flox/flox}$ and *Atg5* $^{flox/flox}$ Lyz-*Cre* mice. Spleen of infected *Atg5* $^{flox/flox}$ Lyz-*Cre* and *Atg5* $^{flox/flox}$ mice was processed for immunohistochemical analysis of ATG5 using specific antibodies directed against ATG5. ATG5 was stained as brown (shown as black arrow heads).

Supplementary FIG 2

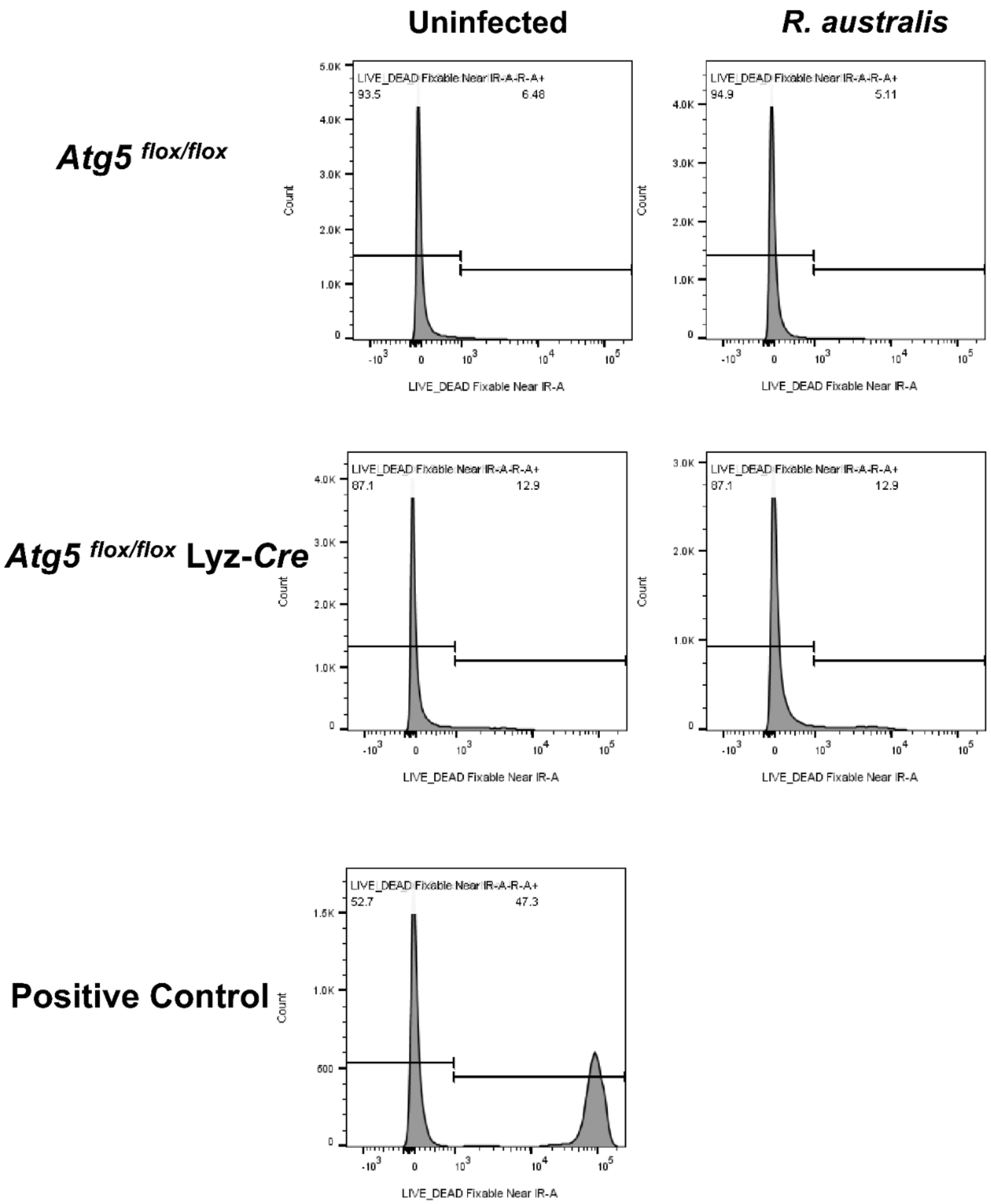

Supplementary FIG 2. Viability of *R. australis*-infected BMMs of *Atg5* $^{flox/flox}$ and *Atg5* $^{flox/flox}$ Lyz-*Cre* mice. BMMs of *Atg5* $^{flox/flox}$ and *Atg5* $^{flox/flox}$ Lyz-*Cre* mice were infected with *R. australis* at an MOI of 2. At 48 h p.i., Cells were collected and stained for viability using LIVE/DEAD® Fixable Near-IR Dead Cell Stain Kit (Life Technologies, Grand Island, NY) in accordance with the manufacturer’s protocol. Positive control was composed of half alive and half dead BMMs of *Atg5* $^{flox/flox}$ Lyz-*Cre* mice. Flow cytometry was performed on 30,000 cells with a FACSCalibur laser cytometer (Becton-Dickinson, BD Biosciences, San Jose, CA). Data analysis on the entire ungated cell population was performed using FlowJo software. Data represented two independent experiments.

Supplementary FIG 3

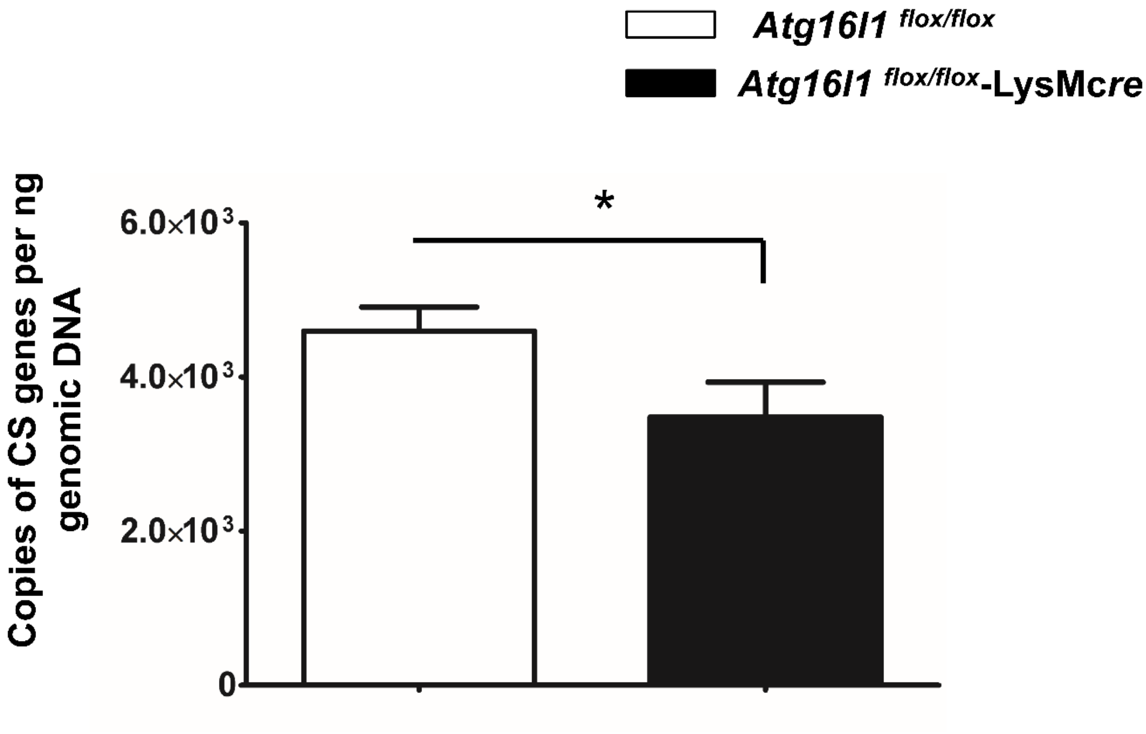

Supplementary FIG 3. Significantly increased concentrations of *R. australis* in *Atg16l1* $^{flox/flox}$ BMMs compared to *Atg16l1* $^{flox/flox}$ -LysM*Cre* BMMs *in vitro*. BMMs of *Atg16l1* $^{flox/flox}$ -LysM*Cre* and *Atg16l1* $^{flox/flox}$ mice were infected with *R. australis* at an MOI of 2. Rickettsial concentration in these primary mouse macrophages was evaluated by quantitative real-time PCR at 48 h p.i.. Number of citrate synthase (CS) gene copies per ng of genomic DNA represents the quantity of rickettsiae. *, p<0.05.
